# Prognostic significance of atrial functional mitral regurgitation in patients with HFpEF and end-stage renal disease

**DOI:** 10.3389/fcvm.2026.1788372

**Published:** 2026-05-15

**Authors:** Yuanyuan Wang, Bingya Lv, Yangyang Chen, Zhe Wang, Botao Hu, Yu Xiao, Haodong Du, Xiaoyi Zhang, Shuai Shao, Ya Suo, Qiankun Bao

**Affiliations:** 1 Tianjin Key Laboratory of Ionic-Molecular Function of Cardiovascular Disease, Department of Cardiology, Tianjin Institute of Cardiology, Second Hospital of Tianjin Medical University, Tianjin, China; 2 National Health Commission Key Laboratory of Cardiovascular Regenerative Medicine, Central China Subcenter of National Center for Cardiovascular Diseases, Henan Cardiovascular Disease Center, Fuwai Central-China Cardiovascular Hospital, Central China Fuwai Hospital of Zhengzhou University, Zhengzhou, China; 3 Department of Kidney Disease and Blood Purification Center, Tianjin Institute of Cardiology, Second Hospital of Tianjin Medical University, Tianjin, China; 4 Department of Kidney Disease and Blood Purification Center, Chifeng Tumor Hospital, Chifeng, China

**Keywords:** AFMR, ANP, ESRD, hemodialysis, HFpEF

## Abstract

**Background:**

Atrial functional mitral regurgitation (AFMR) is common in patients with heart failure with preserved ejection fraction (HFpEF) and end-stage renal disease (ESRD) undergoing hemodialysis, but its prognostic value and potential diagnostic biomarkers remain unclear.

**Methods:**

We conducted a retrospective cohort study involving 452 patients with HFpEF and ESRD undergoing maintenance hemodialysis between August 2018 and September 2023. Clinical outcomes were analyzed using Kaplan–Meier curves and Cox regression. Additionally, 101 patients were prospectively recruited to explore the diagnostic value of plasma atrial natriuretic peptide (ANP) levels for AFMR using ROC analysis.

**Results:**

AFMR was associated with significantly higher all-cause mortality (25.6% vs. 12.3%, *P* = 0.0070). Multivariate Cox regression confirmed AFMR as an independent predictor of all-cause mortality (HR: 2.456; *P* = 0.0090). Even mild AFMR was associated with a higher risk of all-cause mortality (HR: 2.155; *P* = 0.0150). Moderate-severe AFMR was significantly related to a higher incidence of cardiac function deterioration, not only compared with the non-AFMR group (HR: 4.041; 95% CI: 1.186–13.763; *P* = 0.026), but also compared with the mild AFMR group (HR: 3.622; 95% CI: 1.187–11.050; *P* = 0.024). Moreover, ANP levels were significantly elevated in the AFMR group compared to non-AFMR, which could be a significant predictor of AFMR (AUC = 0.73).

**Conclusion:**

AFMR is associated with higher all-cause mortality and worsening cardiac function in patients with HFpEF and ESRD undergoing hemodialysis. ANP may serve as a potential biomarker for diagnosis of AFMR in this patient population. These results underscore the importance of early detection and management of AFMR in this high-risk population.

## GRAPHICAL ABSTRACT

**Figure:**
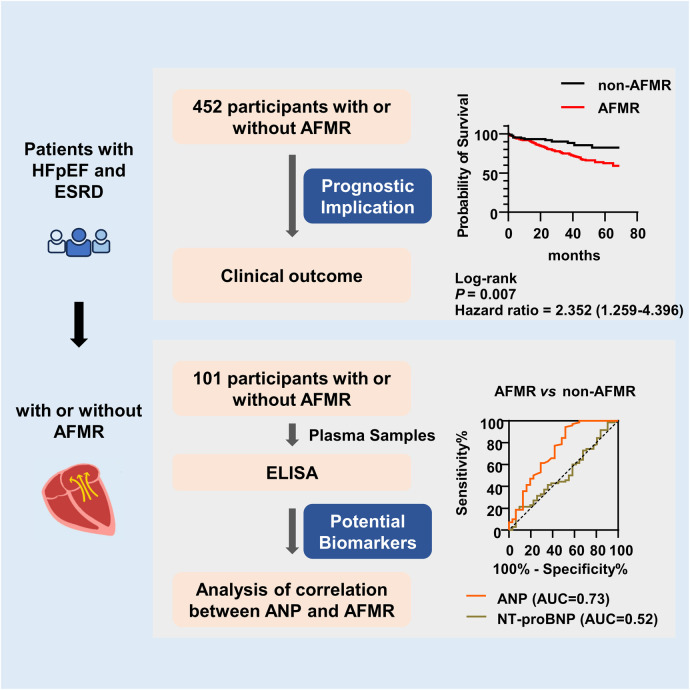
AFMR is associated with increased all-cause mortality and worsening cardiac function in patients with HFpEF and ESRD undergoing hemodialysis. ANP may serve as a potential biomarker for the identification of AFMR in this patient population.

## Introduction

Functional mitral regurgitation (FMR) is primarily associated with abnormal left-sided heart function rather than intrinsic valve pathology (1). A newly recognized subset of FMR, termed atrial functional mitral regurgitation (AFMR), is characterized by mitral annular dilation and left atrial (LA) enlargement, leading to impaired coaptation of the mitral valve leaflets. AFMR typically occurs in the context of heart failure with preserved ejection fraction (HFpEF) or chronic atrial fibrillation, both of which are prevalent in patients with end-stage renal disease (ESRD) undergoing hemodialysis (2, 3). In these patients, left ventricular (LV) diastolic dysfunction can drive atrial remodeling, which contributes to the development of AFMR (4, 5).

Cardiorenal syndromes encompass a range of disorders where cardiac and renal dysfunctions reciprocally exacerbate each other, either acutely or chronically (6, 7). Approximately 30% of patients with chronic kidney disease (CKD) suffer from heart failure (HF), with an annual incidence of 7% among dialysis patients (8). HFpEF, which accounts for over 50% of all HF cases, is particularly prevalent in ESRD and hemodialysis patients, often surpassing the prevalence of heart failure with reduced ejection fraction (HFrEF) (9, 10).

Despite its frequency in clinical practice, AFMR remains an under-recognized and poorly understood condition, with limited therapeutic options and poor prognostic outcomes (11). The ATTEND (Acute decompensated heart failure syndromes) registry found 53% and 18% of 1,825 decompensated patients with HFpEF still showed mild or moderate-severe functional MR at discharge, respectively, even mild FMR has been associated with adverse outcomes in patients hospitalized for acute decompensated heart failure (12). Notably, current guidelines do not address AFMR as a distinct entity, underscoring the need for a better understanding of its pathophysiology and clinical implications. This study aimed to evaluate the prevalence of AFMR and its association with all-cause mortality in patients with HFpEF and ESRD undergoing hemodialysis.

## Methods

### Study design and participants

We conducted the study at the Second Hospital of Tianjin Medical University. The study was approved by the Ethics Committee of the Second Hospital of Tianjin Medical University (No. KY2023K142), and written informed consent was obtained from all participants.

**Figure 1:**
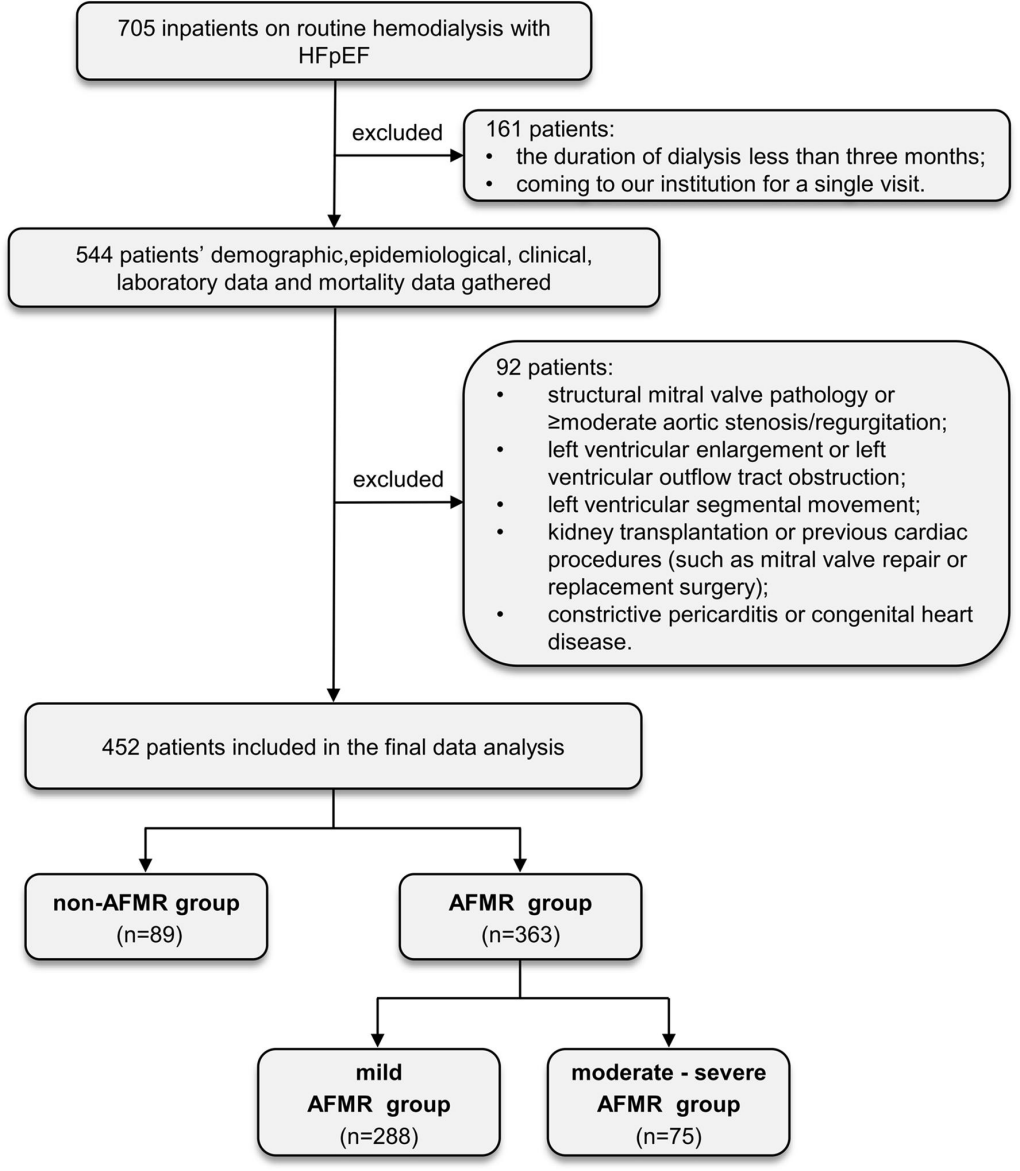
Study design flowchart. 452 patients enrolled in this study underwent data analysis.

We performed a retrospective, single-center cohort study at the Second Hospital of Tianjin Medical University Hemodialysis Center between August 2018 and September 2023. The methods used for the evaluation and management of volume status include assessment of symptom, blood pressure, physical examination, biomarkers, dry weight, and use of bioimpedance (13). Participants were included if they were over 18 years of age and had been diagnosed with HFpEF (LVEF ≥ 50%) and ESRD. According to the latest European Society of Cardiology (ESC) guidelines (14) for the diagnosis of HF, 705 ESRD patients were diagnosed as HFpEF and performed maintenance hemodialysis. Of these, 161 patients were excluded due to the following reasons: dialysis duration of less than three months, a single visit to the institution, or incomplete or unclear echocardiographic data. The remaining 544 patients' demographic, epidemiological, clinical, laboratory data, and mortality data were collected. A further 92 patients were excluded for the following reasons: 1) structural mitral valve pathology or ≥moderate aortic stenosis/regurgitation; 2) left ventricular enlargement or left ventricular outflow tract obstruction; 3) left ventricular segmental motion abnormality; 4) kidney transplantation or previous cardiac procedures (such as mitral valve repair or replacement surgery); and 5) constrictive pericarditis or congenital heart disease. Ultimately, 452 patients were enrolled. According to the 2025 ESC/the European Association for Cardio-Thoracic Surgery (EACTS) Guidelines for the management of valvular heart disease, AFMR is defined by the presence of preserved LVEF (LVEF ≥ 50%) without regional wall motion abnormalities or leaflet tethering, absent or only mild left ventricular dilatation, mitral annular dilatation (anteroposterior diameter >35 mm), and left atrial enlargement (LAVI > 34 mL/m^2^) (15). The patients were categorized into two groups: the non-AFMR group (*n* = 89) and the AFMR group (*n* = 363). According to regurgitant volume, the AFMR group was further sub-grouped into mild AFMR (*n* = 288) and moderate-severe AFMR (*n* = 75). The date of the first echocardiographic diagnosis of the severity of AFMR was chosen as the starting point of the observational period. The flowchart of the study design is shown in Figure 1.

To evaluate biomarkers for AFMR, an additional 101 participants undergoing routine hemodialysis with HFpEF were enrolled from June 15, 2024, to August 31, 2024. The definition of AFMR in the prospective cohort was used the same criteria in the retrospective cohort. In order to avoid hypervolemia that could substantially impact the ANP levels，the blood samples of patients were collected before the second hemodialysis during a mid-week session. According to the transthoracic echocardiography, AFMR was assessed quantitatively (non-AFMR group: *n* = 31, AFMR group: *n* = 70). All participants provided EDTA plasma samples at enrolment, which were stored at −80 ℃ until analysis. Plasma concentrations of atrial natriuretic peptide (ANP) and amino-terminal pro-brain natriuretic peptide (NT-proBNP) were measured using enzyme-linked immunosorbent assays (ELISA).

### Transthoracic echocardiography

All echocardiograms were performed in a single echocardiographic laboratory at the Second Hospital of Tianjin Medical University. Patients on routine hemodialysis (3 times per week, 4 h each) typically underwent transthoracic echocardiography on the mid-week interval day. AFMR was identified by Doppler echocardiography, with digital images and electronic reports reviewed. All images were independently assessed by experienced senior cardiologists who were blinded to the results of other tests. AFMR severity was graded using a standardized and integrative method based on combined qualitative, semiquantitative (vena contracta width), and quantitative evaluation (regurgitant volume). The severity of AFMR was mainly assessed quantitatively by regurgitant volume by continuity equation method performed using standardized methods throughout the study (none: 0 mL, mild: <30 mL, moderate: 30–59 mL, severe: ≥60 mL) (16). The following parameters were evaluated in standard views with standard techniques: left atrium diameter (LAD), left atrium volume (LAV), left ventricular ejection fraction (LVEF), ratio of early diastolic transmitral velocity to early diastolic mitral annular tissue velocity (*E*/*e*′), mitral annular calcification (MAC) and pulmonary artery systolic pressure (PASP). Pulmonary hypertension (PH) was defined as an estimated PASP higher than 35 mmHg at rest. The LAV index (LAVI) was adjusted for body surface area. In patients with AF, echocardiographic parameters (including regurgitant volume) were calculated as the mean of five cardiac cycles. Measurements were performed only in cycles where the preceding and indexed beats were comparable to minimize variability.

### Data collection and study outcome

Epidemiological, demographic, clinical, and laboratory data were extracted from the electronic medical records. Each patient underwent routine laboratory tests before dialysis, including routine blood tests, and kidney function tests. The primary outcome was all-cause mortality. The second outcome was cardiac function deterioration. A reduced EF was defined as an LVEF ≤ 40%, and mildly reduced was defined as EF 41% ≤ LVEF ≤ 49%.

### Statistical analysis

Demographic and clinical characteristics and laboratory indices are presented as mean (standard deviation) or number (percent). Categorical variables were compared between groups using the chi-square test, while continuous variables were compared using one-way ANOVA or a two-tailed *t*-test. Kaplan–Meier curves were plotted for survival analyses, and log-rank tests were used to compare the survival curves for the different groups. Univariate and multivariate analyses of mortality were performed using Cox regression models for death with or without AFMR. The competing-risk model was used to evaluate the association between AFMR and cardiac function deterioration, with all-cause mortality considered as a competing event. The diagnostic values of the parameters were assessed using the receiver operating characteristic (ROC) curve and the area under the ROC curve (AUC). Correlations between regurgitating volume and ANP concentrations were evaluated using the Pearson correlation coefficient (*r*). ANP concentrations were log2 transformed before regression modelling. Then we performed logistic regression analysis to assess the association of ANP levels with AFMR, adjusting for age, sex, diabetes, and hypertension. A *P*-value <0.05 was considered statistically significant. All statistical analyses were performed using the SPSS software (version 26.0) and Stata software (version 15). Graphs were exhibited using GraphPad Prism 9.3.

## Results

### Clinical and echocardiographic characteristics of participants

A total of 452 patients were included in the study, of which 89 were in the non-AFMR group (19.69%) and 363 were in the AFMR group (80.31%). The clinical characteristics of the 452 participants are presented in Table 1. The mean age was 57.1 ± 13.7 years, and 57.7% (261/452) were male. Hypertension and diabetes were the most common comorbidities in dialysis patients. Overall, 87.4% (395/452) of participants had hypertension, 35.6% (161/452) had diabetes, 17.3% (78/452) had a stroke, and 2.4% (11/452) had atrial fibrillation. Regarding the echocardiographic parameters, patients with AFMR had significantly higher LAD, *E*/*e*′, PASP, and tricuspid regurgitation compared to the non-AFMR group, suggesting left atrial enlargement and left ventricular diastolic dysfunction.

**Table 1:** Clinical characteristics of non-AFMR and AFMR patients in the cohort.

	Total (*n* = 452)	non-AFMR (*n* = 89)	AFMR (*n* = 363)	*P* value
Demographic data				
Age (year)	57.1 (13.7)	55.4 (14.8)	57.5 (13.4)	0.2016
Male, *n* (%)	261 (57.7%)	54 (60.7%)	207 (57.0%)	0.5517
Any comorbidity				
Diabetes, *n* (%)	161 (35.6%)	32 (36.0%)	129 (35.5%)	>0.9999
Hypertension, *n* (%)	395 (87.4%)	81 (91.0%)	314 (86.5%)	0.2892
AF, *n* (%)	11 (2.4%)	0 (0%)	11 (3.0%)	0.1323
Prior stroke, *n* (%)	78 (17.3%)	10 (11.24%)	68 (18.7%)	0.1168
Kidney function				
Creatinine (*μ*mol/L)	816.5 (346.7)	792.2 (322.9)	822.9 (352.8)	0.4572
Urea (mmol/L)	26.8 (11.7)	27.8 (13.1)	26.5 (11.3)	0.3535
Medications				
ARNI, *n* (%)	84 (18.6%)	24 (27.0%)	60 (16.5%)	0.0233*
ACEI/ARB, *n* (%)	174 (38.5%)	35 (39.3%)	139 (38.3%)	0.9034
*β*-blocker, *n* (%)	95 (21.0%)	15 (16.9%)	83 (22.9%)	0.2521
CCB, *n* (%)	196 (43.4%)	34 (35.42%)	162 (44.6%)	0.1312
Echocardiographic parameters				
LVEF, (%)	62.3 (4.5)	62.9 (4.4)	62.1 (4.5)	0.1461
LAD, (mm)	40.7 (5.6)	39.3 (5.5)	41.0 (5.6)	0.0096*
IVST, (mm)	10.7 (2.0)	10.9 (2.1)	10.7 (2.0)	0.3996
LVIDd, (mm)	47.9 (4.3)	47.0 (4.5)	48.2 (4.2)	0.0235*
LVPW, (mm)	10.7 (1.9)	10.8 (2.0)	10.6 (1.9)	0.5207
*E*/*e*′	15.2 (6.8)	12.6 (3.9)	15.8 (7.2)	<0.0001*
MAC, (%)	207 (45.8%)	36 (40.5%)	171 (47.1%)	0.2862
PH, *n* (%)	40 (8.8%)	2 (2.2%)	38 (10.5%)	0.0116*
Tricuspid regurgitation, *n* (%)	285 (63.1%)	32 (36.0%)	253 (69.7%)	<0.0001*
Regurgitating volume, (mL)	9.4 (9.4)	0 (0)	15.9 (12.3)	<0.0001*
Vena contracta width, (mm)	1.7 (1.5)	0 (0)	2.1 (1.4)	<0.0001*

Continuous variables are presented as mean (SD), and categorical variables are presented as counts (%). *t* test and chi-square test are used to compare the difference between non-AFMR and AFMR patients. AF, atrial fibrillation; ACEI/ARB, angiotensin converting enzyme inhibitors/angiotonin receptor blocker; CCB, calcium channel blockers; LVEF, left ventricular ejection fraction; LAD, left atrial diameter; LVIDd, left ventricular internal dimension diastole; MAC, mitral annular calcification; AFMR, Atrial Functional Mitral Regurgitation. Multivariable adjusted for: age; diabetes; Prior stroke; NT-proBNP; LVEF, LAD and MAC.

* *P* < 0.05.

### AFMR independently predicts All-cause mortality in patients with HFpEF and ESRD undergoing hemodialysis

To evaluate the relationship between AFMR and all-cause mortality, patients were grouped based on the presence or absence of AFMR. During a median follow-up period of 31 (IQR: 16–48) months, 104 patients experienced all-cause death, including 11 patients in the non-AFMR group (11/89, 12.3%) and 93 patients in the AFMR group (93/363, 25.6%).

**Figure 2:**
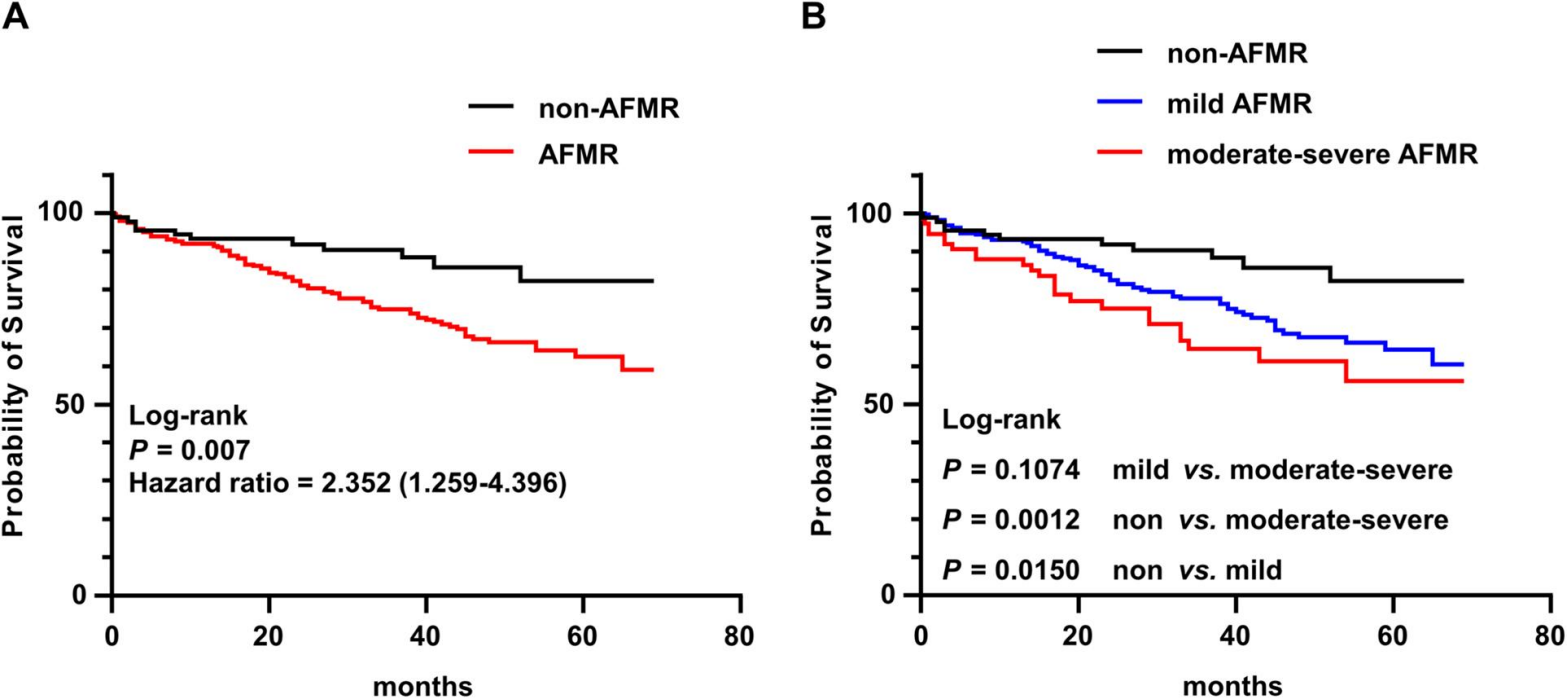
Kaplan–Meier analysis of all-cause death for hemodialysis patients with HFpEF. **(A)** Kaplan–Meier was plotted the survival for hemodialysis patients with HFpEF with (*n* = 363) or without (*n* = 89) functional mitral regurgitation (AFMR). **(B)** Kaplan–Meier was plotted the survival for hemodialysis patients with HFpEF among non-AFMR (*n* = 89), mild AFMR (*n* = 288) and moderate-severe AFMR (*n* = 75).

The Kaplan–Meier curve and log-rank test showed that AFMR were significantly related to an increased incidence of all-cause death and survival rates at 5.75 year were 74% for AFMR patients vs. 88% for non-AFMR patients (HR: 2.352; 95% CI: 1.259–4.396; *P* = 0.0070) (Figure 2A). Furthermore, univariate Cox regression analyses identified age, diabetes, stroke, LVEF, LAD, *E*/*e*′, MAC, and AFMR as factors associated with all-cause mortality in patients with HFpEF and hemodialysis. The multivariate Cox regression analysis showed that AFMR remained independently associated with an increased risk of all-cause mortality, even after adjusting for age, diabetes, stroke, LVEF, LAD, *E*/*e*′, and MAC (HR: 2.456; 95% CI: 1.257–4.796; *P* = 0.009) (Table 2).

**Table 2:** Univariable and multivariable Cox regression analysis of risk factors for all-cause mortality.

Variable	Univariable		Multivariable	
	HR (95% CI)	*P* value	HR (95% CI)	*P* value
Age	1.043 (1.027–1.060)	<0.0001	1.032 (1.014–1.051)	0.0006
Sex	1.244 (0.835–1.855)	0.2833	Not selected	
Diabetes	1.672 (1.138–2.458)	0.0088	1.410 (0.949–2.095)	0.0890
Hypertension	0.629 (0.357–1.107)	0.1080	Not selected	
AF	1.755 (0.714–4.314)	0.2206	Not selected	
Prior stroke	1.665 (1.084–2.557)	0.0199	1.207 (0.763–1.909)	0.4209
Creatinine	1.000 (0.999–1.000)	0.1446	Not selected	
Urea	0.997 (0.980–1.014)	0.7302	Not selected	
ARNI	0.857 (0.494–1.485)	0.5814	Not selected	
ACEI/ARB	0.992 (0.670–1.467)	0.9662	Not selected	
β-blocker	0.698 (0.423–1.149)	0.1574	Not selected	
CCB	0.998 (0.678–1.469)	0.9926	Not selected	
LVEF	0.964 (0.925–1.005)	0.0824	0.966 (0.925–1.009)	0.1200
LAD	1.039 (1.004–1.075)	0.0290	1.005 (0.968–1.044)	0.7867
IVST	1.026 (0.934–1.127)	0.5960	Not selected	
LVIDd	0.987 (0.946–1.030)	0.5491	Not selected	
LVPW	1.029 (0.930–1.138)	0.5804	Not selected	
*E*/*e*′	1.018 (0.997–1.040)	0.0989	0.991 (0.956–1.028)	0.6252
MAC	2.540 (1.680–3.840)	<0.0001	1.650 (1.049–2.594)	0.0302
PH	1.563 (0.902–2.709)	0.1115	Not selected	
Tricuspid regurgitation	1.307 (0.922–1.852)	0.1328	Not selected	
AFMR	2.352 (1.259–4.396)	0.0073	2.456 (1.257–4.796)	0.0085

AF, atrial fibrillation; ACEI/ARB, angiotensin converting enzyme inhibitors/angiotonin receptor blocker; CCB, calcium channel blockers; LVEF, left ventricular ejection fraction; LAD, left atrial diameter; LVIDd, left ventricular internal dimension diastole; MAC, mitral annular calcification; AFMR, Atrial Functional Mitral Regurgitation. Multivariable adjusted for: age; diabetes; Prior stroke; NT-proBNP; LVEF, LAD and MAC.

### The association between severity of AFMR and mortality risk

The regurgitating volume was the main measure parameter used to classify mitral regurgitation severity. Based on the severity of AFMR, patients were sub-grouped into mild AFMR and moderate-severe AFMR. The clinical characteristics of the participants are presented in Table 3. In the moderate-severe AFMR group, the percentage of patients with AF was significantly higher compared with both the non-AFMR group and mild AFMR group, reflecting a potential relationship between atrial fibrillation and AFMR. Regarding the echocardiographic parameters, patients with moderate-severe AFMR had significantly higher LAD, *E*/*e*′, and PASP than those in the non-AFMR and mild AFMR groups, indicating left atrial enlargement and left ventricular diastolic dysfunction. Kaplan–Meier analysis showed that even mild AFMR was associated with an increased risk of all-cause death in patients with HFpEF and hemodialysis and survival rates at 5.75 year were 76% for mild AFMR patients vs. 88% for non-AFMR patients (HR: 2.155; 95% CI: 1.304–3.561; *P* = 0.015). Patients with moderate-severe AFMR had a somewhat higher risk of all-cause death than those with mild AFMR, but the difference was not statistically significant (HR: 1.530; 95% CI: 0.912–2.568; *P* = 0.107) (Figure 2B).

**Table 3:** Clinical characteristics of non-AFMR, mild AFMR and moderate-severe AFMR patients in the cohort.

Variable	non-AFMR (*n* = 89)	mild AFMR (*n* = 288)	moderate-severe AFMR (*n* = 75)
Demographic data			
Age (year)	55.4 (14.8)	56.7 (13.4)	60.8 (13.2)
Male, *n* (%)	54 (60.7%)	164 (56.9%)	43 (57.3%)
Any comorbidity			
Hypertension, *n* (%)	81 (91.0%)	246 (85.4%)	68 (90.7%)
Diabetes, *n* (%)	32 (36.0%)	99 (34.4%)	30 (40.0%)
Prior stroke, *n* (%)	10 (11.2%)	52 (18.1%)	16 (21.3%)
AF, *n* (%)	0 (0%)	2 (0.7%)	9 (12.0%)^#^^,^^†^
Kidney function			
Creatinine (μmol/L)	792.2 (322.9)	826.6 (365.1)	810.6 (301.1)
Urea (mmol/L)	27.8 (13.1)	26.4 (11.6)	27.1 (10.2)
Medications			
ARNI, *n* (%)	24 (27.0%)	49 (17.0%)	11 (14.7%)
ACEI/ARB, *n* (%)	35 (39.3%)	106 (36.8%)	33 (44.0%)
β-blocker, *n* (%)	15 (16.9%)	64 (22.2%)	19 (25.3%)
CCB, *n* (%)	34 (35.4%)	123 (42.7%)	39 (52.0%)
Echocardiographic parameters			
LVEF, (%)	62.9 (4.4)	62.5 (4.5)	60.5 (4.0)^#^^,^^†^
LAD, (mm)	39.3 (5.5)	39.5 (4.3)	47.1 (5.9)^#^^,^^†^
IVST, (mm)	10.9 (2.1)	10.6 (1.9)	11.0 (2.2)
LVIDd, (mm)	47.0 (4.5)	47.8 (4.1)	49.6 (4.5)^#^^,^^†^
LVPW, (mm)	10.8 (2.0)	10.5 (1.8)	11.0 (2.2)
*E*/*e*′	12.6 (3.9)	15.1 (7.1)*	18.7 (6.9)^#^^,^^†^
MAC, (%)	36 (40.5%)	130 (45.1%)	41 (54.7%)
PH, *n* (%)	2 (2.2%)	18 (6.3%)	20 (26.7%)^#^^,^^†^
Tricuspid regurgitation, *n* (%)	32 (36.0%)	192 (66.7%)*	61 (81.3%)^#^^,^^†^
Regurgitating volume, (mL)	0 (0)	10.5 (6.2)*	36.8 (5.2)^#^^,^^†^
Vena contracta width, (mm)	0 (0)	1.6 (0.6)*	4.4 (1.1)^#^^,^^†^

Continuous variables are presented as mean (SD), and categorical variables are presented as counts (%). Chi-square test and one-way ANOVA test are used to compare the difference among non-AFMR, mild AFMR and moderate—severe AFMR patients. non-AFMR vs. mild AFMR. non-AFMR vs. moderate-severe AFMR; mild AFMR vs. moderate—severe AFMR. AF, atrial fibrillation; ACEI/ARB, angiotensin converting enzyme inhibitors/angiotonin receptor blocker; CCB, calcium channel blockers; LVEF, left ventricular ejection fraction; LAD, left atrial diameter; LVIDd, left ventricular internal dimension diastole; MAC, mitral annular calcification; AFMR, Atrial Functional Mitral Regurgitation. Multivariable adjusted for: age; diabetes; Prior stroke; NT-proBNP; LVEF, LAD and MAC.

* *P* < 0.05.

# *P* < 0.05.

† *P* < 0.05.

### Moderate-severe AFMR is associated with progressive decline in cardiac function

To observe the changes in cardiac function among the three groups at the endpoint of follow-up, we collected the echocardiography and clinical data until last follow-up and performed a comparative analysis with the initial cardiac function. During follow-up, cardiac function was reduced in 31 patients, including 6 patients in the non-AFMR group [6/89, 6.7%; HFmrEF (6)], 14 patients in the mild AFMR group [14/288, 4.9%; HFmrEF (12); HFrEF (2)], and 11 patients in the moderate-severe AFMR group [11/75, 14.7%; HFmrEF (5); HFrEF (6)].

**Figure 3:**
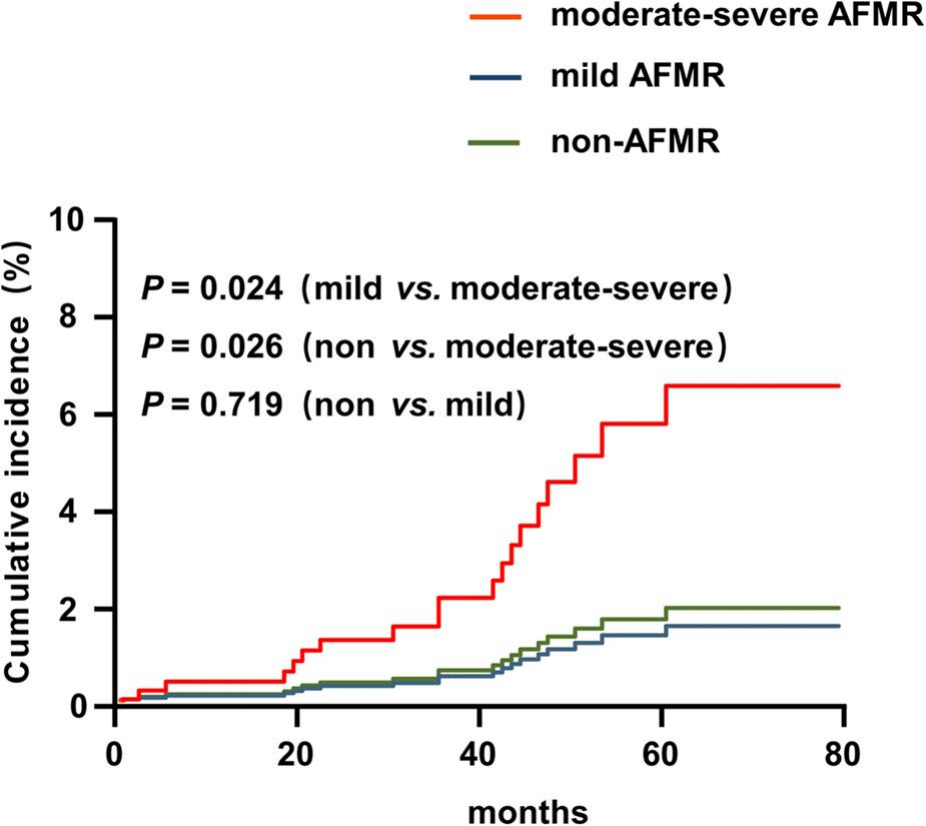
Competing risk model analysis of heart failure (LVEF ≤ 49%) for hemodialysis patients with HFpEF. The model estimates the heart failure (LVEF ≤ 49%) for hemodialysis patients with HFpEF (*n* = 452) among non-AFMR, mild AFMR and moder-severe AFMR groups with considering death as a competing risk.

Given the high mortality in this cohort, we used a competing-risk model to evaluate cardiac function deterioration. After adjusting for age, sex, comorbidities (diabetes, hypertension, atrial fibrillation and stroke), kidney function and medications, moderate-severe AFMR was still significantly associated with increased risk of cardiac function deterioration compared with the non-AFMR group (HR: 4.041; 95% CI: 1.186–13.763; *P* = 0.026) and the mild AFMR group (HR: 3.622; 95% CI: 1.187–11.050; *P* = 0.024). No significant difference was observed between non-AFMR and mild AFMR (HR: 0.786; 95% CI: 0.211–2.925; *P* = 0.719) (Figure 3).

### Potential diagnostic value of ANP for AFMR

Currently, AFMR diagnosis is performed via transthoracic echocardiography, but patients on long-term hemodialysis typically do not have regular cardiac ultrasounds. However, they do have routine blood tests. Therefore, we investigated whether a blood biomarker could help identify AFMR. An additional 101 participants undergoing routine hemodialysis with HFpEF were enrolled and grouped into the non-AFMR group (*n* = 31) and AFMR group (*n* = 70). The clinical characteristics are presented in Table 4.

**Table 4:** Clinical characteristics of non-AFMR and AFMR patients in the cohort for biomarker.

Variable	non-AFMR (*n* = 31)	AFMR (*n* = 70)	*P* value
Demographic data			
Age, (year)	54.0 (12.2)	54.4 (12.7)	0.8871
Male, *n* (%)	14 (45.2%)	37 (52.9%)	0.5224
BMI (kg/m^2^)	25.1 (3.9)	23.9 (4.0)	0.1716
Any comorbidity			
Diabetes, *n* (%)	4 (12.9%)	18 (25.7%)	0.1953
Hypertension, *n* (%)	25 (80.7%)	64 (91.4%)	0.1800
AF, *n* (%)	1 (3.2%)	0 (0%)	0.3069
Prior stroke, *n* (%)	5 (16.1%)	6 (8.6%)	0.3054
Medications			
ARNI, *n* (%)	6 (19.4%)	21 (30.0%)	0.3337
ACEI/ARB, *n* (%)	11 (35.5%)	28 (40.0%)	0.8250
β-blocker, *n* (%)	4 (12.9%)	17 (24.3%)	0.2881
CCB, *n* (%)	13 (41.9%)	34 (48.6%)	0.6660
Laboratory examination			
WBC (×10^9^/L)	6.1 (1.4)	6.4 (2.7)	0.5618
NEU (×10^9^/L)	4.1 (1.1)	4.5 (2.5)	0.4220
Haemoglobin (g/L)	108.9 (10.9)	108.0 (11.7)	0.7195
Platelet (×10^9^/L)	197.9 (54.4)	201.7 (59.3)	0.7589
Urea (mmol/L)	31.7 (14.07)	26.9 (8.9)	0.0762
Creatinine (μmol/L)	969.4 (343.4)	902.4 (326.5)	0.4279
eGFR (mL/min/1.73m^2^)	3.4 (1.1)	3.5 (1.1)	0.8867
Uric acid (μmol/L)	382.1 (110.1)	360.8 (99.7)	0.3402
Calcium (mmol/L)	2.2 (0.3)	2.3 (0.2)	0.1874
Phosphorus (mmol/L)	2.3 (0.6)	2.2 (0.7)	0.5291
ANP (pg/mL)	314.5 (231.9)	503.4 (268.7)	0.0010*
NT-proBNP (pg/mL)	3,323 (5,184)	4,607 (7,053)	0.3713
Echocardiographic parameters			
LAD, (mm)	38.9 (4.1)	41.0 (4.9)	0.0403*
LAV, (mm^3^)	48.7 (14.5)	56.6 (19.0)	0.0427*
LAVI	49.9 (16.2)	58.6 (16.8)	0.0171*
LVEF, (%)	62.0 (3.5)	61.9 (3.8)	0.8902
IVST, (mm)	9.9 (1.5)	10.3 (1.9)	0.2550
LVIDd, (mm)	46.1 (5.1)	47.5 (5.0)	0.2006
LVIDs, (mm)	26.7 (3.7)	27.4 (4.8)	0.4517
LVPW, (mm)	9.7 (1.4)	10.3 (2.0)	0.1272
*E*/*e*′	11.9 (4.1)	15.3 (5.3)	0.0022*
MAC, (%)	12 (38.7%)	24 (65%)	0.0334*
PH, *n* (%)	0 (0%)	5 (12.5%)	0.0661
Regurgitating volume, (mL)	0 (0)	17.7 (11.4)	<0.0001*
Vena contracta width, (mm)	0 (0)	2.0 (1.0)	<0.0001*

Continuous variables are presented as mean (SD), and categorical variables are presented as counts (%). *t* test and chi-square test are used to compare the difference between non-AFMR and AFMR patients. AF, atrial fibrillation; ACEI/ARB, angiotensin converting enzyme inhibitors/angiotonin receptor blocker; CCB, calcium channel blockers; LVEF, left ventricular ejection fraction; LAD, left atrial diameter; LVIDd, left ventricular internal dimension diastole; MAC, mitral annular calcification; AFMR, Atrial Functional Mitral Regurgitation. Multivariable adjusted for: age; diabetes; Prior stroke; NT-proBNP; LVEF, LAD and MAC.

* *P* < 0.05.

**Figure 4:**
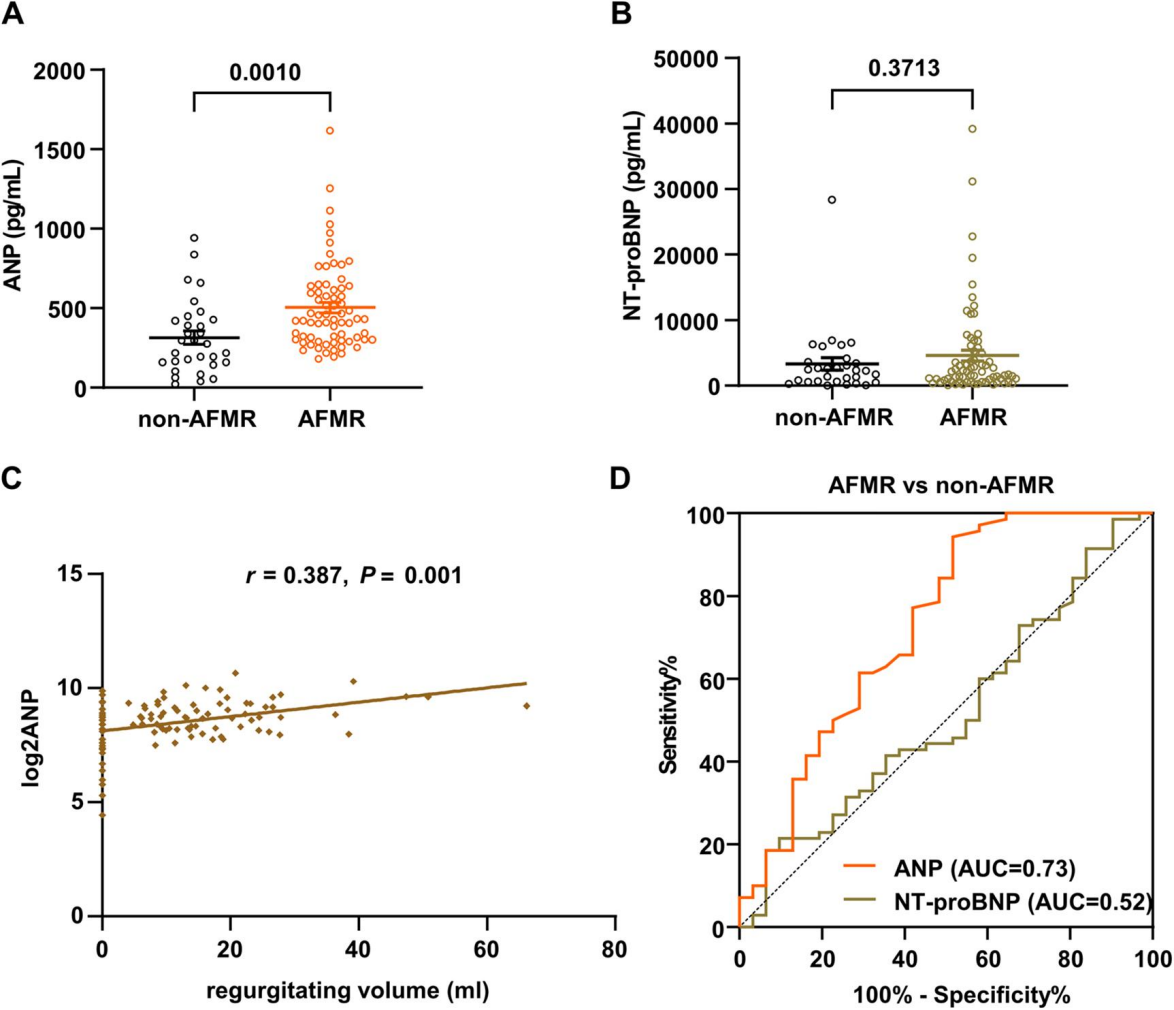
ANP and NT-proBNP levels in non-AFMR and AFMR patients and receiver operating characteristic (ROC) curves in the validation cohort. **(A,B)** Levels of ANP and NT-proBNP in the non-AFMR (*n* = 31) and AFMR (*n* = 70) groups were analyzed. Significance differences were assessed using two-tailed *t*-tests. **(C)** Correlations between ANP level and regurgitating volume in hemodialysis patients with HFpEF. The ANP level (*r* = 0.387) is positively correlated with regurgitating volume. **(D)** ROC curves of plasma levels of ANP and NT-proBNP in the non-AFMR (*n* = 31) and AFMR (*n* = 70) groups.

Levels of ANP were significantly higher in the AFMR group compared to the non-AFMR group, while NT-proBNP levels did not differ significantly between groups (Figures 4A,B). Echocardiographic parameters showed that the AFMR group had significantly higher LAD, LAV, LAVI, and *E*/*e*′ than the non-AFMR group, indicating left atrial enlargement and diastolic dysfunction. Left ventricular function and structure parameters were not significantly different between groups (Table 4). Furthermore, after adjusting for demographic factors (age, sex, and BMI) and comorbidities (diabetes, hypertension, atrial fibrillation, and stoke), ANP was significantly correlated with regurgitating volume (*r* = 0.387; *P* < 0.001) (Figure 4C).

To estimate the diagnostic value of ANP for AFMR, we calculated the AUC. ANP showed an AUC of 0.73, which is considered acceptable diagnostic accuracy, while NT-proBNP had a lower AUC of 0.52. Univariate logistic regression analysis indicated that ANP (>225.5 pg/mL) was a potential biomarker for associated with AFMR (OR = 1.004; 95% CI: 1.001–1.006) (Figure 4D).

## Discussion

The present study provides several important findings regarding the prevalence, prognostic significance, and early diagnosis for AFMR in patients with HFpEF and ESRD undergoing hemodialysis. Firstly, our study demonstrates that AFMR is highly prevalent in this high-risk population. Secondly, we found that AFMR was associated with increased all-cause mortality, highlighting the poor prognosis of these patients. Finally, our analysis indicates that ANP may serve as a potential marker of atrial dysfunction that could prompt further echocardiographic investigation.

AFMR has been documented in HFpEF patients, although the number of studies is limited. One study found moderate-severe AFMR in 63% of HFpEF patients, with most having mild mitral regurgitation (4, 17). In our study, 80.3% of HFpEF patients undergoing hemodialysis had AFMR, with 79.3% having mild MR. Compared to those without AFMR, patients with AFMR had higher rates of PH, LA dilation, and elevated *E*/*e*′ ratios, all indicators of left ventricular diastolic dysfunction. Previous observational studies have reported an AF prevalence of 13%–27% among ESRD patients with a mean age of 60–67 years (18, 19). The significantly lower incidence of AF (2.4%) in our study compared to other studies. AF in our study was identified solely by electrocardiogram at a single enrollment timepoint, without continuous rhythm monitoring, which may have led to underestimation of its prevalence, particularly in paroxysmal AF or cases managed with rhythm control.

During the follow-up period, moderate-severe AFMR was significantly related to an increased incidence of reduced cardiac function. Indeed, the causes of death in patients with ESRD are largely attributable to heart failure, often with HFpEF, and related sudden cardiac death (20). Several mechanisms may explain the association between AFMR and poor outcomes in patients with HFpEF and ESRD. First, FMR in HF patients typically arises without intrinsic mitral valve pathology, driven by altered forces on the mitral leaflets. This results in increased LV filling pressure, pulmonary venous hypertension, and LA dilation, all of which are associated with mortality in patients with preserved EF (4). Hypertension and diabetes, common comorbidities in dialysis patients, are associated with different patterns of LA remodeling, including more severe atrial dilation and higher storage capacity in those with both HFpEF and ESRD compared to HFpEF alone. In summary, AFMR imposes a dual volume load on the LA and LV, leading to progressive dilation of both chambers and a consequent imbalance between LA and LV volumes. This volumetric mismatch disrupts atrioventricular coupling and impairs efficient forward flow, ultimately resulting in a reduction in effective stroke volume. Second, we found that patients with moderate-severe AFMR had a significantly higher incidence of PH than those in the non-AFMR and mild AFMR groups. Finally, abnormal calcium-phosphorus metabolism in chronic renal failure directly contributes to MAC formation (21). Our data show that 47.1% of AFMR patients had MAC. The association of MAC with increased cardiovascular mortality is well-documented.

The patients on long-term hemodialysis usually don't have regular echocardiography for AFMR unless they have cardiac symptoms, but they do have regular blood test. Given the association between AFMR and poor outcomes, identifying reliable biomarkers for early diagnosis is critical. In patients with CKD and HF, sodium retention, fluid overload, and increased vascular tension stimulate the release of biomarkers such as NPs (22). Our study found that ANP levels were significantly higher in the AFMR group compared to the non-AFMR group, while NT-proBNP levels did not differ significantly between the groups. ANP is released in response to atrial wall stretch, which occurs in AFMR due to LA enlargement, offering a valuable tool for early diagnosis.

Increased LA volume index emerged as the independent echocardiographic determinant of mortality in dialysis ESRD patients treated by strict volume control (23). The LA structural and functional changes that may enhance the risk of AFMR. Guideline-directed medical therapy (GDMT) remains the cornerstone of FMR management (24, 25). However, no North American or European guidelines specifically address AFMR as a distinct entity (26, 27). In our cohort, patients with AFMR had a significantly lower percentage of ARNI usage than non-AFMR patients (Table 1, *P* = 0.023). It remains unclear whether lower ARNI usage in the AFMR group could partly explain their worse outcomes. Adoption of medical therapies for HF recommended by the guidelines provides promising results to favor LA reverse remodeling (28). In the PRIME trial (Pharmacological Reduction of Functional, Ischemic Mitral Regurgitation), the LA volume index improved significantly with ARNI therapy (29). However, a limitation of most studies, including PRIME, is that they did not differentiate between atrial and ventricular FMR. Further studies are needed to evaluate whether ARNI or SGLT2 inhibitors can improve outcomes by reducing AFMR severity.

### Limitations

This study has several inherent limitations due to its retrospective design. It was conducted at a single center with a relatively small sample size and a short follow-up period, which may introduce selection bias. These suggest the need for larger, prospective studies to better understand the impact of AFMR on patients. Additionally, the standardization of volume control and the measures of hemodialysis adequacy were not fully factored, fluid control during dialysis may significantly influence left ventricular end-diastolic pressure, potentially leading to AFMR as a secondary effect. Finally, due to its cross-sectional design, the association between ANP and AFMR in the present study does not suggest the casual relationship. Further study is required to establish the direct role of biomarkers.

## Conclusions

In patients with HFpEF and ESRD, AFMR is common and significantly associated with adverse outcomes. Our study demonstrates that the presence of AFMR is associated with increased all-cause mortality and decline in cardiac function. Even mild AFMR is associated with a higher risk of death compared to patients without AFMR. Therefore, echocardiographic assessment for AFMR may have value in risk stratification, even among asymptomatic individuals. As routine follow-up in this population relies more on laboratory testing than imaging, the observed elevation of ANP (but not NT-proBNP) in AFMR suggests a potential role for ANP in identifying patients who may warrant further echocardiographic evaluation. These findings underscore the prognostic value of AFMR in this high-risk population and highlight the need for early identification and potential targeted management strategies.

## Data Availability

The original contributions presented in the study are included in the article/Supplementary Material, further inquiries can be directed to the corresponding author.
